# Sustained high HIV case‐finding through index testing and partner notification services: experiences from three provinces in Zimbabwe

**DOI:** 10.1002/jia2.25321

**Published:** 2019-07-19

**Authors:** Nyikadzino Mahachi, Auxilia Muchedzi, Taurayi A Tafuma, Peter Mawora, Liz Kariuki, Bazghina‐werq Semo, Moses H Bateganya, Tendai Nyagura, Getrude Ncube, Mike B Merrigan, Otto N Chabikuli, Mulamuli Mpofu

**Affiliations:** ^1^ FHI 360 Harare Zimbabwe; ^2^ FHI 360 Washington DC USA; ^3^ FHI 360 Durham NC USA; ^4^ FHI 360 Pretoria South Africa; ^5^ U.S. Agency for International Development Harare Zimbabwe; ^6^ Ministry of Health and Child Care Harare Zimbabwe

**Keywords:** index testing, partner notification services, HIV positivity, HIV, Zimbabwe

## Abstract

**Introduction:**

Several countries in southern Africa have made significant progress towards reaching the Joint United Nations Programme on HIV/AIDS goal of ensuring that 90% of people living with HIV are aware of their status. In Zimbabwe, progress towards this “first 90” was estimated at 73% in 2016. To reach the remaining people living with HIV who have undiagnosed infection, the Zimbabwe Ministry of Health and Child Care has been promoting index testing and partner notification services (PNS). We describe the implementation of index testing and PNS under the Zimbabwe HIV Care and Treatment (ZHCT) project and the resulting uptake, HIV positivity rate and links to HIV treatment.

**Methods:**

The ZHCT project has been implemented since March 2016, covering a total of 12 districts in three provinces. To assess the project's performance on index testing, we extracted data on HIV testing from the district health information system (DHIS 2) from March 2016 to May 2018, validated it using service registers and calculated monthly HIV positivity rates using Microsoft Excel. Data were disaggregated by district, province, sex and service delivery point. We used SPSS to assess for statistical differences in paired monthly HIV positivity rates by sex, testing site, and province.

**Results:**

The average HIV positivity rate rose from 10% during the first six months of implementation to more than 30% by August 2016 and was sustained above 30% through May 2018. The overall facility HIV positivity rate was 4.1% during the same period. The high HIV positivity rate was achieved for both males and females (mean monthly HIV positivity rate of 31.3% for males and 33.7% for females), with females showing significantly higher positivity compared to males (*p* < 0.001). The ZHCT mean monthly HIV positivity rate from index testing (32.6%) was significantly higher than that achieved through provider‐initiated testing and counselling and other facility HIV testing modalities (4.1%, *p* < 0.001).

**Conclusions:**

The ZHCT project has demonstrated successes in implementing index testing and PNS by attaining a high HIV positivity rate sustained over the study period. As the country moves towards HIV epidemic control, index testing and PNS are critical strategies for targeted HIV case identification.

## 1 Introduction

HIV testing and counselling is the first crucial step towards achieving the Joint United Nations Programme on HIV/AIDS (UNAIDS) 90‐90‐90 targets 1, where 90% of people living with HIV (PLHIV) know their HIV status, 90% of those diagnosed with HIV receive sustained antiretroviral therapy (ART) and 90% of those on ART achieve viral suppression. Results from population‐based HIV impact assessments completed in 10 countries show that southern African countries have made significant progress towards achieving these targets, although HIV case‐finding continues to be a challenge 2. In 2016, 86% and 74% of PLHIV in Eswatini and Zimbabwe, respectively, knew their HIV status 2, compared to Tanzania and Uganda which had achieved 52% and 62% of the first 90, respectively. Findings also suggest a higher HIV case‐finding gap amongst males compared to females. In Zimbabwe, for example, 77% of females and 69% of males living with HIV are aware of their status 2.

Since the onset of the HIV epidemic, countries in sub‐Saharan Africa have introduced and expanded several HIV testing modalities. Voluntary HIV counselling and testing (VCT), where individuals voluntarily seek HIV testing services, has been a cornerstone of HIV diagnosis in southern Africa 3. Community VCT reaches a higher proportion of clients who received their first HIV test compared to standard clinic‐based VCT 4. In health facilities, provider‐initiated testing and counselling has been a key service for promptly identifying PLHIV and prioritizing their care 5, 6, 7. Home‐based, mobile and outreach testing, though costly, have also been important modalities for reaching men, young adults, and key populations 8, 9, 10.

Despite the expansion in HIV testing services, some countries in sub‐Saharan Africa have not been able to reach the first 90 goal. To address this gap, several countries have introduced index testing services in both facility and community settings. The World Health Organization defines index testing as a focused HIV testing approach in which the partners and biological children of people diagnosed with HIV are offered HIV testing services 11. PNS involves asking HIV‐positive individuals (known as index cases) to list their sex partners, then contacting and offering all listed partners an HIV test and linking all partners diagnosed as HIV‐positive to ART 12. Index testing and PNS have emerged as targeted and effective strategies in identifying previously undiagnosed individuals in sub‐Saharan Africa and other settings. Models for index testing vary by country. In Tanzania, index testing is implemented by contacting and testing partners of index clients identified after a positive HIV test 13. In Malawi, index testing follows a patient already enrolled in HIV treatment services who reports having an untested household member 14, and in Cameroon, children (ages 0 to 19 years) of an adult living with HIV are tested for HIV at approved treatment centres, and siblings of any of the children who test positive are also subsequently tested. 15. PNS has also been successfully used in the control of sexually transmitted infections in the United States and sub‐Saharan Africa 16, 17, 18, 19, 20.

In the 1990s, concerns about increased risk of domestic violence and a lack of widespread and affordable ART caused delays in the introduction and adoption of index testing and PNS in HIV programs 21, 22. However, recent implementation of targeted index testing and PNS has shown high HIV positivity rates in Uganda (32%) 23, Cameroon (50.1%) 24 and Malawi (54%) with few reports of adverse events 25.

The Zimbabwe HIV Care and Treatment (ZHCT) project, funded through the U.S. Agency for International Development, has adopted index testing and PNS as part of its community HIV testing programme. In this paper, we describe (1) the implementation and uptake of index testing and PNS in three provinces in Zimbabwe; (2) HIV positivity rates by month, province, sex and type of contact; and (3) linkage rates to ART after an HIV‐positive diagnosis.

## 2 Methods

### 2.1 Study setting

**Figure 1:**
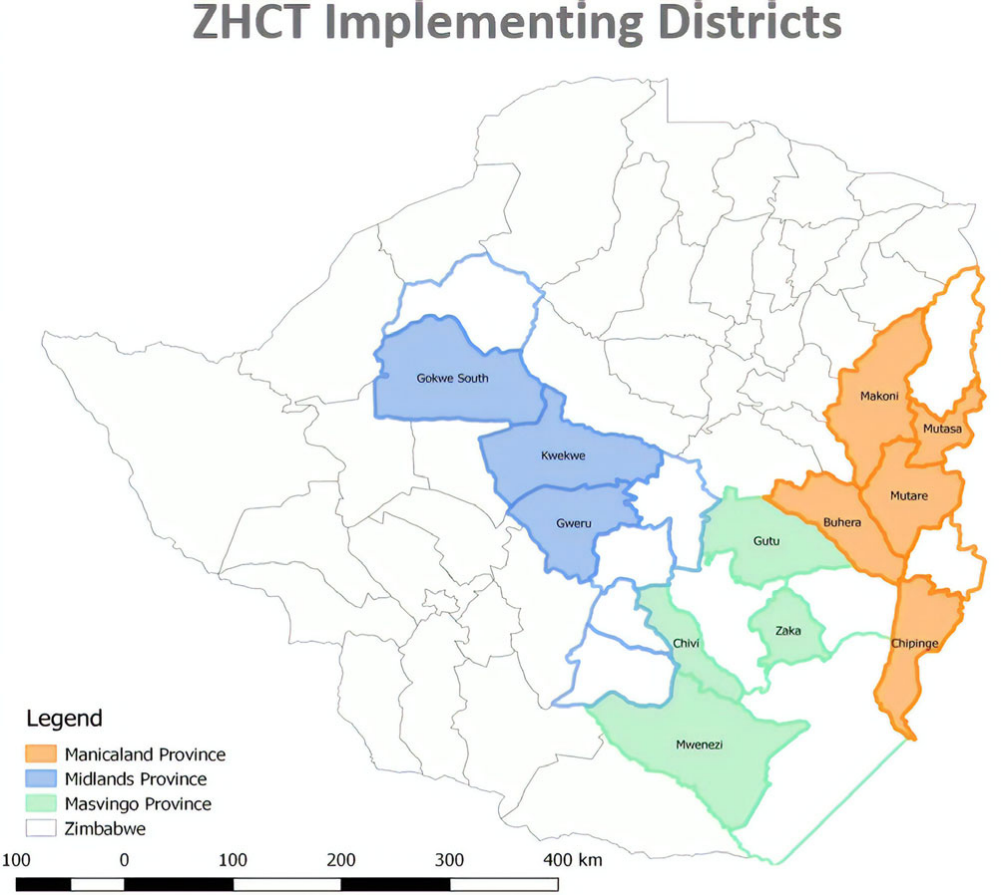
Distribution of the 12 ZHCT‐supported districts in three provinces

ZHCT is a community HIV testing project implemented in 12 districts (Figure 1) across three rural provinces of Zimbabwe. These provinces are Manicaland, with an adult HIV prevalence of 11.4%; Midlands, with a prevalence of 14.1%; and Masvingo, with a prevalence of 14.9% 26.

The combined estimated population of the 12 districts is 3,039,865 27, and ART coverage is approximately 87.2% 26. All 12 districts have been prioritized by the U.S. President's Emergency Plan for AIDS Relief (PEPFAR) and the Government of Zimbabwe for HIV epidemic control by 2020. PEPFAR defines HIV epidemic control as the point at which the total number of new infections falls below the total number of deaths from all causes among PLHIV 28.

### 2.2 Target population

The project provides HIV testing services to partners (sexual contacts) and biological children of index clients as per World Health Organization 11 and national guidelines. For ZHCT, index clients are defined as individuals who are newly or recently (within six months) diagnosed as HIV‐positive and registered at one of the 317 public‐sector health facilities (three provincial hospitals, 12 district hospitals, 302 clinics) within the 12 districts.

### 2.3 ZHCT index testing and PNS procedures

**Figure 2:**
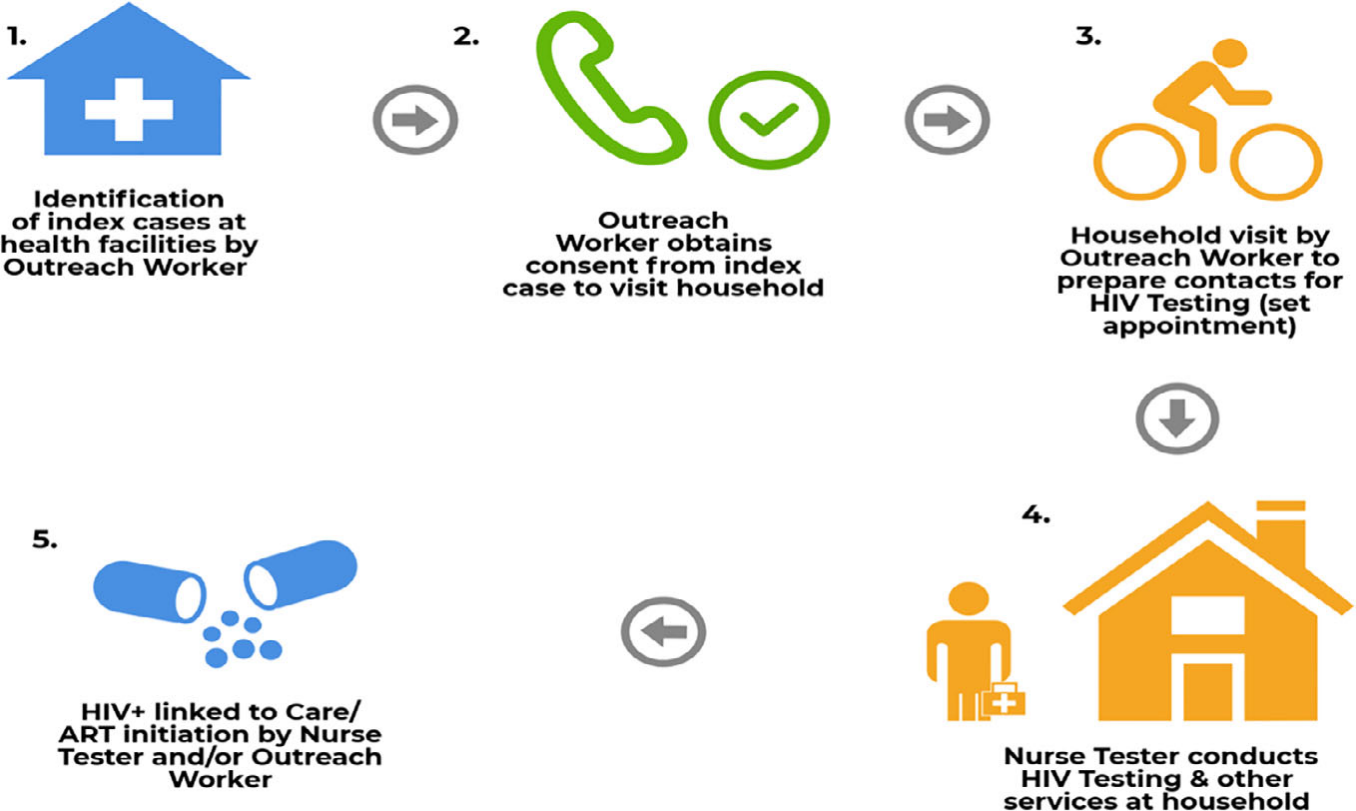
ZHCT index testing and PNS process flowchart

Nurses in public‐sector facilities, and ZHCT‐supported community nurse testers and outreach workers, collaborate closely to offer index testing and PNS (Figure 2).

Facility‐based nurses provide HIV testing services to health facility clients. Community nurse testers and outreach workers (expert patients) who have been stable and adherent to medication for at least five years visit health facilities in their catchment area every weekday, review facility registers, and identify newly or recently diagnosed HIV‐positive (index) clients. The nurse testers or outreach workers then contact the index clients to obtain consent for PNS, assuring clients of the confidentiality of the testing services. When requested by the index clients, nurse testers support disclosure and provide couples counselling services.

The outreach workers make appointments for a home visit and prepare the index clients’ contacts for HIV testing. During the scheduled visits at home (or other mutually agreed upon location), community nurse testers conduct rapid HIV testing and counselling for all partners and biological children for whom consent has been obtained. Rapid HIV testing is done as per the Zimbabwe national guidelines.

In addition to being tested for HIV, partners and children are screened for non‐communicable diseases, such as hypertension (blood pressure screening) and diabetes mellitus (random blood sugar); malnutrition; tuberculosis; and sexually transmitted infections. Community nurse testers also discuss family planning options and distribute condoms. Clients who test HIV positive in the community are referred and/or escorted to the nearest health facility or facility of preference for enrollment into ART services. Additionally, clients who test positive or have abnormal findings during other screenings are referred for appropriate care to the nearest facility capable of providing the necessary health care services.

The project employs 45 community nurse testers and 264 outreach workers. Each nurse tester is responsible for providing the services mentioned above across five health facilities and the surrounding catchment area, and each outreach worker is responsible for supporting one to two health facilities. All nurse testers are trained and certified in rapid HIV testing through competency‐based assessments, and they participate in proficiency testing and external quality assurance once a year. The project also conducts a five‐day orientation programme for newly‐recruited nurse testers, which covers information on service delivery models of community HIV testing (including index testing and PNS), index case identification and risk assessments, linkage and ART initiation, quality assurance, infection control, disposal of biological waste, and implementation fidelity. The outreach workers receive project‐specific training for five days upon recruitment. Their training includes modules on community mobilization, household engagement for index and PNS services, outreach workers’ roles and responsibilities, documentation and data collection tools, linkage to care and treatment for individuals who test HIV positive and tracking and testing of sexual partners of index cases. Outreach workers also receive on‐the‐job mentorship and supervision from field‐based clinical staff employed by the project and an annual three‐day refresher course.

### 2.4 Data collection and analysis

District aggregate HIV testing data were extracted from the Ministry of Health and Child Care's DHIS‐2 database by project monitoring and evaluation officers, while ZHCT programme data were extracted from the project's DHIS‐2 database and imported into Microsoft Excel. All aggregate data were validated using HIV testing services summary reports for both community and facility‐based HIV testing. The HIV positivity rate, defined as the percentage of newly diagnosed people living with HIV amongst the total tested during the same period, was the main descriptive statistic/indicator of interest. Additional variables included number of index clients registered and newly diagnosed contacts linked to ART services after an HIV positive test. HIV positivity results were assessed by age group, relationship of contact to index client, province and sex.

To assess the sustained high HIV positivity over time, rates were calculated by month. To demonstrate efficiency in HIV testing within ZHCT, we assessed the percentage contribution of ZHCT's community index testing and PNS to the overall number of HIV tests provided and positive cases identified by month in each district. We also compared ZHCT's HIV positivity rate with that of facility‐based testing by month.

Additionally, paired t‐tests were used to test for statistical significance in mean difference between the monthly HIV positivity rates of pairs matched for sex (males and females); place of HIV test (facility‐based testing and community index testing); and provinces. HIV positivity rates were considered paired between sex, place of test, and provinces because these rates were obtained by month. Rates by group (e.g., male and female) were obtained for each month from May 2016 through May 2018 leading to 13 matched‐paired values for each comparison. SPSS Version 24 (IBM SPSS Statistics for Windows, Version 24.0.; IBM Corp., Armonk, NY) was used to analyze for statistical differences in HIV positivity between different parameters. A *p* ≤ 0.05 was used to indicate a statistically significant difference in mean monthly HIV positivity for assessed parameters.

### 2.5 Ethical considerations

The ZHCT project received a non‐research determination from the Medical Research Council of Zimbabwe (MRCZ number MRCZ/E/159).

## 3 Results

**Figure 3:**
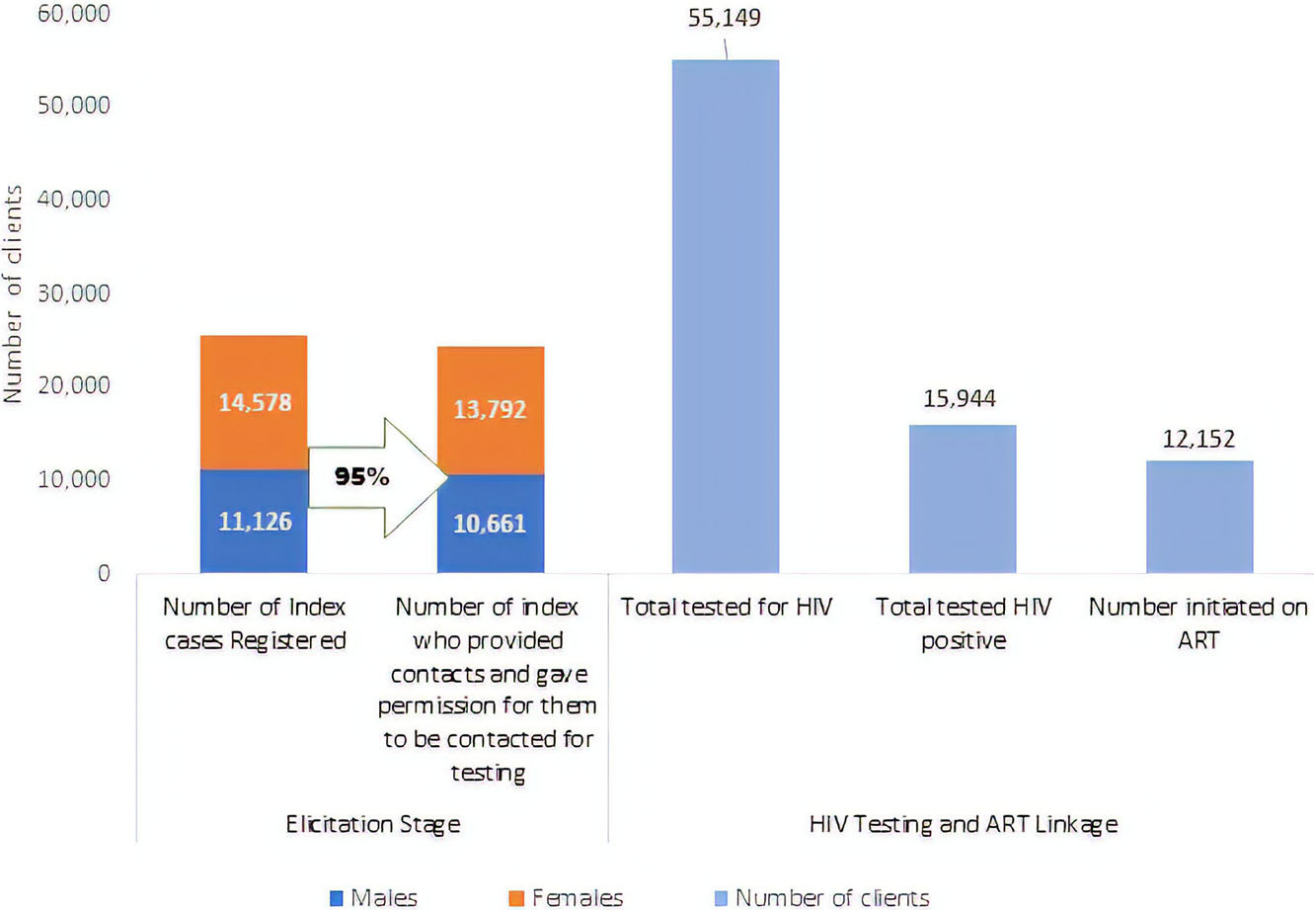
ZHCT index testing cascade for the 12 districts in Manicaland, Midlands and Masvingo provinces, March 2016–May 2018

From March 2016 to May 2018, the ZHCT project registered 25,704 index clients, of whom 24,453 (95.1%) consented to participate in index testing and PNS. These clients provided 55,149 contacts who received HIV testing services. Of these 55,149 contacts, 15,944 (29%) tested HIV positive. Figure 3 shows the index testing and PNS cascade for the 12 districts during this period.

The HIV positivity rate was highest amongst contacts who were 45 to 49 years old, with a positivity rate of 43% (Table 1). Sexual partners of index clients had an HIV positivity rate of 46% compared to children under the age of 15 at 17%. Amongst the provinces, Manicaland had the lowest positivity rate of 20%, while Midlands had the highest at 41%. The HIV positivity for females was 30% compared to 33% for males. The HIV positivity rates also differed significantly for different age groups, relationship to index case, provinces and sex (*p* < 0.001) (Table 1).

**Table 1:** HIV positivity by age group, relationship to index client, province, and sex, March 2016–May 2018 (n = 55,149)

	Total tested for HIV	Total tested HIV positive	% HIV positivity	*p* value^a^
Age group (years)				
<5	2006	215	10.7	<0.001
5 to 9	1384	231	16.7	
10 to 14	1381	208	15.1	
15 to 19	4183	626	15.0	
20 to 24	8005	1883	23.5	
25 to 29	8903	2624	29.5	
30 to 34	9353	2962	31.7	
35 to 39	7197	2720	37.8	
40 to 44	4894	1924	39.3	
45 to 49	2660	1136	42.7	
50+	5183	1415	27.3	
Total	55,149	15,944	28.9	
Relationship to index case				
Sexual partner	24,099	11,115	46.1	<0.001
Child	3463	575	16.6	
Other (relatives in the household)	27,562	4254	15.4	
Province				
Manicaland (n = 5 districts)	26,582	5195	19.5	<0.001
Midlands (n = 3 districts)	21,064	8645	41.0	
Masvingo (n = 4 districts)	7503	2104	28.0	
Sex				
Male	26,468	7253	27.4	<0.001
Female	28,681	8691	30.3	

a *p* value for Chi Square test that the HIV positivity is the same for disaggregates in each characteristic.

### 3.1 Sustaining high HIV positivity over time

**Figure 4:**
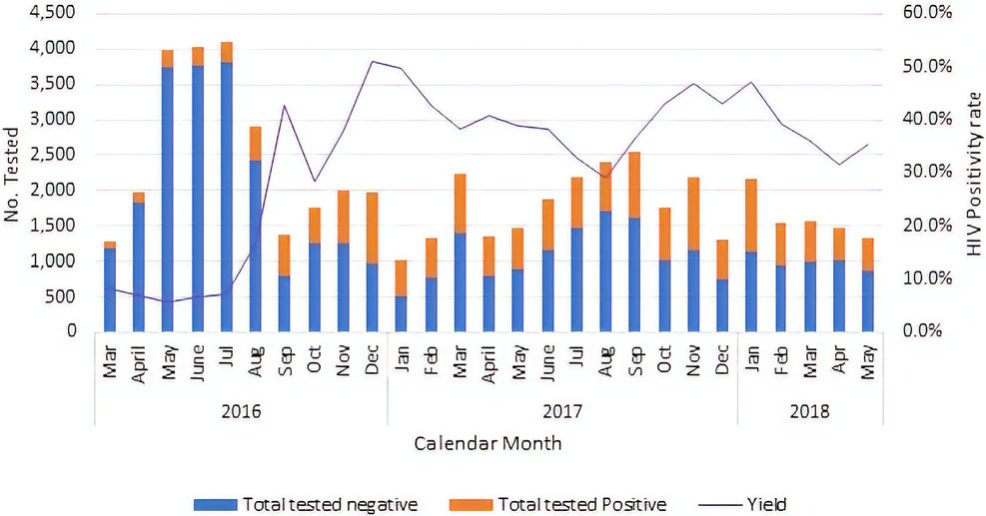
Number of clients tested within the ZHCT project through index testing and PNS, and percent HIV positive by calendar month, March 2016–May 2018

Figure 4 illustrates monthly HIV positivity rates for two distinct time periods: (1) March to August 2016, when the project was testing a high volume of clients, resulting in HIV detection rates of less than 10% and (2) August 2016–2018, when HIV positivity surpassed 30% after a shift in strategy from household testing to only testing sexual partners and biological children of index clients. HIV positivity rates remained high during the 21 months of the study period with a peak in positivity (>50%) noted in December 2016.

**Figure 5:**
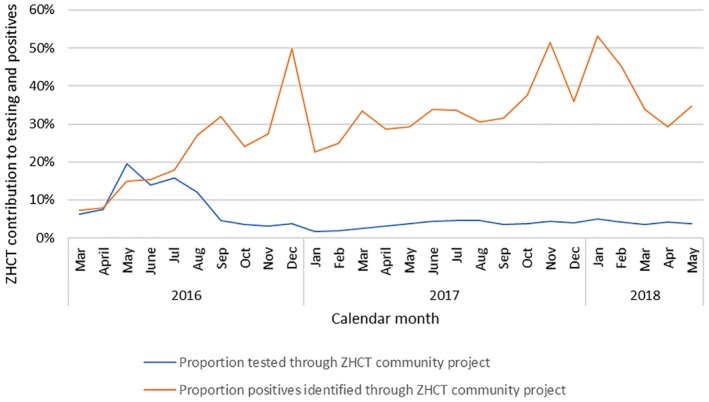
Percentage contribution of ZHCT community index testing and PNS to overall HIV tests and positive cases identified by month, March 2016–May 2018

Analysis of ZHCT project data in comparison with the overall district‐level data extracted from the DHIS‐2 database shows that before Oct 2016, ZHCT was contributing less than 20% of both clients tested and cases identified. From September 2016, when community index testing and PNS were strengthened, the overall contribution to the total number tested reduced to less than 5%, while the contribution to the total cases identified increased to more than 30% and reached a high of 54% in January 2018 (Figure 5). During the study period, 1,264,642 patients were tested for HIV and 46,033 of these were HIV positive.

**Figure 6:**
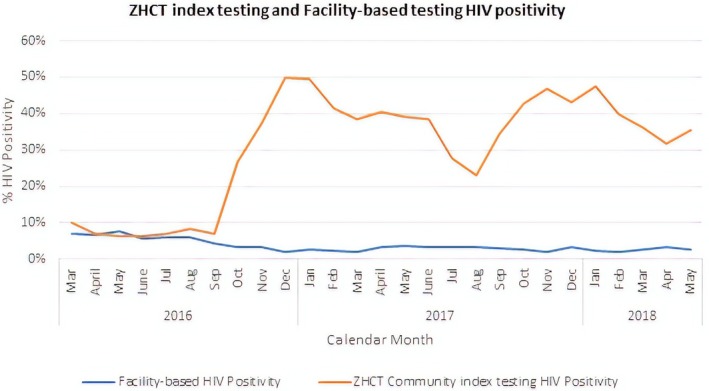
Comparison of HIV positivity rates between ZHCT and facility‐based testing in 12 districts in Zimbabwe, March 2016–May 2018

ZHCT's HIV positivity rate from community index testing and PNS was at least five times greater than that achieved by health facilities within the same districts (Figure 6).

At the start of the ZHCT project between March and August 2016, the project's HIV positivity was comparable with facility‐based testing at less than 10%. However, after the introduction index testing and PNS, the HIV positivity rate increased to at least 30% in the next 21 months with facility‐based testing remaining consistently below 5% every month.

Paired t‐test results of data matched for place of test, sex and province showed a statistically significant difference in mean monthly HIV positivity of 28.5% between ZHCT community index testing and PNS compared to facility‐based testing (*p* < 0.001), as well as a higher HIV positivity rate of 2.4% among women compared to men (*p* < 0.001) (Table 2).

**Table 2:** Difference in mean monthly HIV positivity

Matched pairs	Difference in mean monthly HIV positivity (%)	t‐statistic	*p*‐value
ZHCT positivity/facility positivity	28.5	9.408	<0.001
Females/males (ZHCT)	2.4	5.363	<0.001
Midlands Province/Manicaland Province (ZHCT)	24.3	9.020	<0.001
Midlands Province/Masvingo Province (ZHCT)	31.1	9.798	<0.001
Manicaland Province/Masvingo Province (ZHCT)	−1.7	‐0.805	0.433

Among the provinces, the Midlands had a significantly higher HIV positivity rate than both Manicaland and Masvingo (*p* < 0.001). Masvingo had a higher mean monthly positivity yield than Manicaland, although this was not statistically significant (*p* = 0.433).

## 4 Discussion

The ZHCT project demonstrated success in attaining and sustaining high HIV case‐finding through index testing and partner notification services. During the first six months of the project, the proportion of HIV‐positive individuals identified was low and comparable to HIV detection rates at surrounding health facilities. The low HIV positivity rate of less than 10% is explained by the fact that the start‐up phase served as a learning phase. Low HIV positivity was recorded initially due to poor targeting by the nurse testers. As the project generated data, learned from it, and made improvements in programming, the need for a shift in testing strategy became apparent. The shift from household testing of every family member to testing only sexual partners and biological children reduced the volume of clients tested and resulted in an increase in the HIV positivity rate.

As donor funding continues to decline in resource‐limited countries, efficiency in testing becomes even more critical. Index testing has been widely implemented and prioritized by all provinces and districts and is accepted by health providers within ZHCT. This testing modality is also consistent with PEPFAR recommendations on scaling up effective and efficient HIV testing strategies leading to fewer people being tested but more HIV‐positive cases being identified 29. Partner testing services have achieved high HIV positivity rates in pilot studies; for example, 32% positivity was achieved among HIV‐exposed individuals in high‐risk populations in Uganda 23; 23% positivity in a randomized control trial in Kenya 12; and 62% HIV positivity in Tanzania 30. ZHCT provides a programmatic example of how to effectively implement index testing and PNS at scale, targeting the general population, and successfully achieving and sustaining high HIV positivity rates.

PNS has been known as an effective HIV case‐finding strategy for population groups at high risk of HIV for many years 31. However, early concerns related to perceived high costs 32, fears of gender‐based violence and reduced effectiveness related to partner anonymity and stigma hindered its scale‐up 33. Over time, these concerns have been slowly addressed, and index testing has proved to be one of the best means for identifying HIV‐positive individuals in sexual networks, thus helping to interrupt HIV transmission 34, 35. Partner notification data offer the best opportunity to elucidate risk networks and their configurations 36, 37. While index testing and PNS have only recently been promoted within HIV services in sub‐Saharan Africa, these have widely been used for almost two decades in Western countries 38, 39, 40, 41.

In Zimbabwe, ZHCT pioneered the implementation of index testing and PNS. The project achieved high HIV positivity rates regardless of clients’ sex, although the positivity amongst females was marginally but significantly higher than males. The high positivity rate amongst both sexes is not surprising because the services target sexual contacts and young children (less than 15 years) of HIV‐positive index clients. There was also no significant difference in uptake of PNS between males and females within ZHCT, which differs from findings in Tanzania where males were 2.2 times more likely than female index clients to get at least one sexual partner to come in for HIV testing 13. We interpreted this positively, because testing programs struggle to find men and index testing may address the HIV case‐finding gap among men.

While HIV positivity was high across all districts, rates in the districts in the Midlands province (Kwekwe and Gokwe) were close to 60%. Kwekwe is a mining town that also has a high concentration of artisanal mining activity around it, dominated by young single men, and Gokwe has a thriving agriculture production and trading sector. High HIV prevalence is well documented to be one of the biggest threats to the mining workforce in developing countries 42. Indeed, Zimbabwe's artisanal minors are known to have a high HIV prevalence 43, in part because of having high numbers of sexual partners and more frequent contact with female sex workers in their settlements 44. Similarly, productive and trade agriculture locations are also characterized by a young mobile population with high rates of sex work, which increases risks of HIV transmission 45. Many population groups in Kwekwe and Gokwe district also have poor health‐seeking behaviors. Index testing and PNS may help reach people who do not traditionally seek health care.

According to the latest Zimbabwe population‐based HIV impact assessment (ZIMPHIA), 73% of PLHIV are diagnosed and 87% of those diagnosed are on ART 26. Reaching the remaining undiagnosed PLHIV will require targeted testing modalities. ZHCT achieved HIV positivity rates above 30% with index testing and PNS services. In contrast, facility‐based testing achieved positivity rates of less than 5% and contributed less than 60% of HIV‐positive clients identified, despite testing more than 95% of all clients. The significant progress that has been made towards the 90‐90‐90 goals in most sub‐Saharan African countries has made finding the few remaining undiagnosed HIV‐positive individuals a challenge, and reliance on traditional facility‐based testing is no longer adequate nor sustainable. A decade ago, home‐based and facility‐based VCT were achieving HIV positivity rates as high as 56% and 26%, respectively 46. With more people tested and aware of their HIV status, such broad strategies are no longer effective on their own. Scale‐up of index testing and PNS is necessary to target HIV testing services and reach the remaining undiagnosed PLHIV 29. While the focus is scaling up index testing and PNS, it is worth noting that to a large extent, these services in Zimbabwe are dependent on facility‐based testing from which index clients are identified, making the two equally important and complementary.

As is typical of using programme data, there were some limitations. First, the data analyzed for this manuscript were disaggregated by age groups, but these age bands were not disaggregated by sex. Doing so could have provided a more precise picture of the performance of index testing and PNS, particularly amongst specific population groups such as adolescent girls and young women and middle‐aged men who are at high risk of HIV but have proved challenging to identify in HIV programs. The data could also not be disaggregated by urban versus rural areas because of the overlap in districts that contained both rural and urban settlements. Additionally, data on number of elicited contacts and those offered HIV testing were not readily available, so we could not assess the number of refusals following offer of HIV testing services. Moreover, the project continues to evolve by integrating additional services, such as screening for noncommunicable diseases. Including these data would have helped to share outcomes of ZHCT index testing and PNS beyond HIV case identification; however, these data were not available because the services started a few months before the study. We were also unable to share HIV positivity rates from other testing modalities such as VCT and outreach testing for the same period because these data are not available in the DHIS 2 database. Nonetheless, this manuscript provides important lessons to other programs in the process of implementing index testing and PNS.

## 5 Conclusions

Community index testing and PNS have been successful in identifying undiagnosed PLHIV in Zimbabwe and achieving high HIV positivity rates. The ZHCT project has sustained testing positivity above 30% over the period of implementation, demonstrating that PNS are feasible, efficient and effective. As the country makes significant progress towards achieving HIV epidemic control, these strategies will be increasingly critical in reaching specific high‐risk populations and need to be implemented in conjunction with other effective HIV testing strategies.

## Competing interests

The authors declare that they have no competing interests.

## Authors’ contributions

NM and AM led the conceptualization of the paper and led the writing of the methods section. PM and TAT led the extraction, validation and cleaning of the programme data and contributed towards data analysis and the writing of the results and methods section. MM led the data analysis, and writing of the introduction, results and discussion. BS and MHB wrote part of the introduction, the methods, and results, and LK contributed to writing the methods section. MBM, ONT, TN and GN reviewed all sections of the manuscript. All authors contributed to interpreting the data and editing of the manuscript and all approved the final version.
